# Comparative Evaluation of Antibacterial Efficacy of Emblica Officinalis Lollipop Against Streptococcus Mutans Counts in Institutionalized Visually Impaired Children

**DOI:** 10.7759/cureus.28207

**Published:** 2022-08-20

**Authors:** Sphurti P Bane, Nilima R Thosar, Nilesh V Rathi, Meghana A Deshpande, Pranjali V Deulkar

**Affiliations:** 1 Pediatric and Preventive Dentistry, Private Practitioner, Mumbai, IND; 2 Pediatric and Preventive Dentistry, Sharard Pawar Dental College and Hospital, Datta Meghe Institute of Medical Sciences, Wardha, IND; 3 Pediatric Dentistry, Dr. D. Y. Patil Dental College and Hospital, Dr. D. Y. Patil Vidyapeeth, Pimpri, IND; 4 Pediatric and Preventive Dentistry, Private Practitioner, Delhi, IND; 5 Pediatric and Preventive Dentistry, Private Practitioner, Nagpur, IND

**Keywords:** emblica officinalis, herbal lollipop, children with special needs, visually impaired, dental care for children

## Abstract

Background

Among the 1.21 billion population of India, 26.8 million individuals have disabilities, and around five million are visually impaired. These children encounter problems related to oral health maintenance, thus further leading to dental problems. Even though Pediatric dentists treat visually impaired children for their oral problems, they emphasize mainly on the prevention of dental caries. Dental caries has a multifactorial etiology, and dentists are unable to find a complete solution for its prevention. However, reducing *Streptococcus mutans *has been seen to reduce the caries rate in the past. The use of the herbal product *Emblica officinalis* to reduce *Streptococcus mutans *levels has been documented. An affordable delivery system is required to use *Emblica officinalis* for its anticaries action. Thus, a unique delivery system of herbal sugar-free lollipops containing *Emblica officinalis* extract was made and can effectively deliver antimicrobial action in visually impaired children.

Aim

To evaluate the antibacterial efficacy of *Emblica officinalis* lollipop on *Streptococcus mutans *counts and pH levels in institutionalized visually impaired children.

Method

A total of 60 institutionalized visually impaired children (age: 4 to 14 years) were selected. The study consisted of two groups (experimental “*Emblica officinalis* lollipop” and control “placebo lollipop”), and the children were divided equally into both groups. Children from the study and control groups were subjected to the respective lollipops twice daily for seven days. *Streptococcus mutans* count and pH count were evaluated at baseline and after seven days post-intervention of the respective lollipop.

Results

The results showed that in both groups, *Streptococcus mutans* count was reduced post-intervention. However, the efficacy of the study group (*Emblica officinalis* lollipop) in inhibiting the *Streptococcus mutans* count was better than the control group (placebo lollipop). An increase in the pH level was seen post-intervention for both the study and control groups. And on the intergroup comparison, no statistical significance was found.

Conclusion

The use of *Emblica officinalis* lollipop is effective in inhibiting the *Streptococcus mutans* count when compared with the placebo lollipop. While marginal pH change was seen in both groups. Thus, the herbal modality most acceptable without any pharmaceutical concerns should be chosen. *Emblica officinalis* lollipops can be used in institutionalized visually impaired children to reduce the oral *Streptococcus mutans* count and maintain a healthy oral cavity.

## Introduction

The American Health Association has defined a disabled child as one who cannot fully use all of his physical, mental, and social abilities. Amongst the 26.8 million disabled population of India, nearly two million comprise children. Of all the disabilities, nearly 0.15% of the children are visually impaired [1]. The presence of visual impairment affects not just the general health but also the oral health of an individual. One of the reasons for such a finding can be the poor psychomotor development of the individual [2].

Literature suggests a high incidence of dental caries and periodontal diseases in visually impaired children owing to their lack of hand-eye synchronization and inadequate parental supervision [3-5]. Additionally, factors such as difficulty in diagnosis, poor compliance, financial constraints, and lack of awareness in society suggest that providing preventive compared to therapeutic services to this sector of the population will be more beneficial [6].

The use of herbal products in dentistry has been increasing in the past few decades in the form of intracanal irrigants, medicaments, and obturating materials. Among the home care remedies, herbal mouthwash, toothpaste, gel, gums, and candies have gained popularity recently. Herbal lollipops are a newer entity introduced to aid in the reduction of microbial colonization. The majority of these contain *Glycyrrhiza uralensis* (liquorice) [7-11]. However, the presence of glycyrrhizin present in liquorice root has been known to cause muscle weakness, headache, blurred vision, and an increase in blood pressure [12].

*Emblica officinalis *(Amla/Indian Gooseberry) is a well-acclaimed drug in phytodentistry. It is known for its antibacterial, antifungal, and antiviral properties and antioxidant activity [13]. Hasan S et al., through their study, mentioned *Emblica officinalis* extracts to be better than the pure compounds (phthalic acid and furfuraldehyde) against *Streptococcus mutans* [14]. Thus, the present study was planned to evaluate the antibacterial efficacy of *Emblica Officinalis* lollipops against *Streptococcus mutans *in institutionalized visually impaired children.

## Materials and methods

The study synopsis was approved, and official permissions were obtained from the Institutional Ethics Committee (IEC) before commencing the study (IEC/2018-19/7523). This was an interventional type of study conducted on visually impaired children of Wardha district, Maharashtra, India. The complete study model was explained to the Institution head as well as parents/guardians of the children to be included in the study, and written informed consent was obtained from them.

Inclusion and exclusion criteria

Institutionalized visually impaired children in age group 4-14 years residing in Wardha district with total blindness and systemically healthy were recruited for the study. Visually impaired children having any systemic medical conditions or who were on long-term antibiotics, or those undergoing comprehensive dental treatments in the past four weeks were excluded from the study. Children with mild, moderate, or severe blindness were also excluded from the study.

Study design & sample size calculation

**Figure 1:**
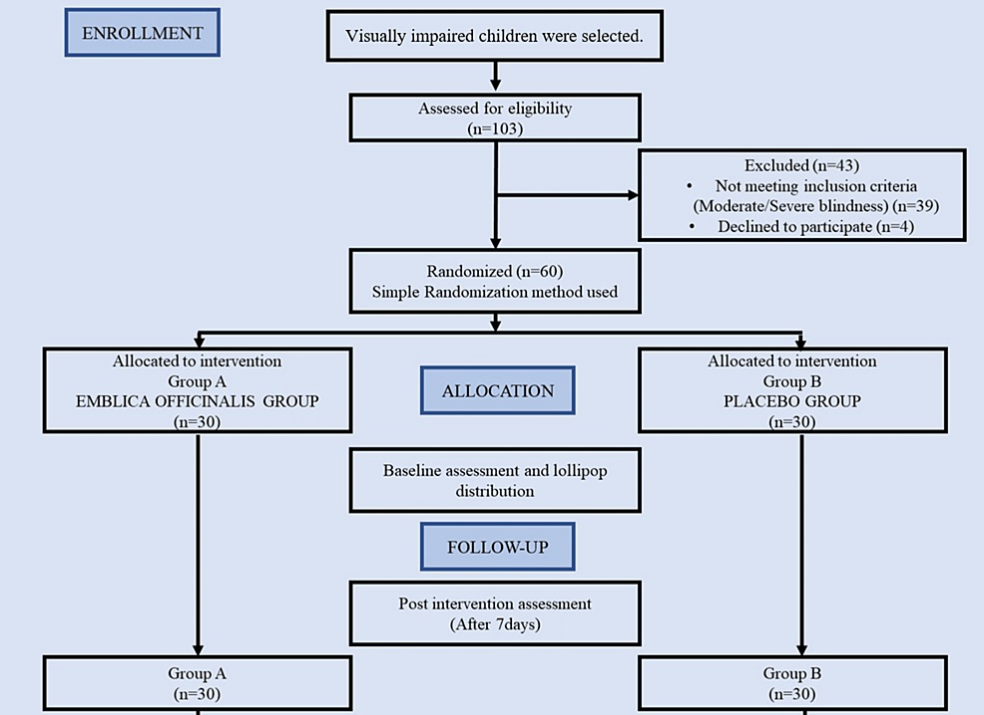
Flow chart of the study The figure is created by the author.

The present study was planned as a double-blinded, randomized, interventional type of study. The sample size was calculated using 95% probability, showing a statistically significant difference using an alpha level and power of 80% by nMaster software (Tamil Nadu, India) and using the mean and standard deviation formula. According to the software, the sample size calculated was 27, but considering the possible attrition in the study's future, 27 children were rounded off to 30 children in each group. These children were further randomly divided into two groups by one examiner: Group A (n=30) and Group B (n=30). The study flowchart is depicted in Figure 1.

Preparation of lollipops

**Figure 2:**
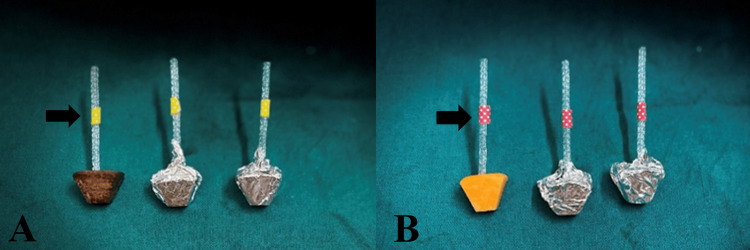
Colour coded lollipops A: Yellow-coded lollipops are* Emblica officinalis* lollipops; B: Red-coded lollipops are placebo lollipops The figure is created by the author.

The *Emblica officinalis* lollipops were prepared in collaboration with the Institute of Pharmaceutical Education and Research, Wardha, India, and are registered with the Copyright Office, Government of India (L-100887/2021). The primary content of these lollipops was trehalose and *Emblica officinalis *extract. The herbal extract was obtained from a registered ayurvedic vendor. To 100 ml of *Emblica officinalis* extract, 70% w/v of trehalose was added along with 20% w/v of isomalt powder and dissolved. The mixed solution was then heated at 50˚C in a hot air oven till 3/4th volume was evaporated. After that, mannitol (3% w/v of trehalose) was added, and evaporation was continued until 5% of the solution’s original volume remained. The hot thick mixture was further poured into the molds and cooled to room temperature for complete solidification. The *Emblica officinalis* lollipops were retrieved on solidification, wrapped separately, and stored at room temperature. Placebo lollipops were prepared similarly to *Emblica officinalis* lollipops except without *Emblica officinalis* content. The lollipops used in the study were color-coded (Figure 2).

The *Emblica officinalis* lollipop was coded with yellow tape, while the placebo lollipop was marked with red tape and handed over to the caregivers. The caregivers were not aware of the lollipop content and its color coding.

Distribution of lollipops

The children were told to continue their use of fluoridated toothpaste and soft bristle brush during the present study. Verbal oral hygiene instructions were given to the children before the study commenced and was practiced under the caregiver’s supervision just as an additional reinforcement to their daily oral hygiene regime. The lollipops were given twice daily for seven consecutive days. The caregivers were instructed to distribute the lollipops to children post meals according to their allocated groups (For Group A: Yellow tape lollipops, and for Group B: Red tape lollipops). The children were instructed to consume it completely and hand over the sticks back to the caregivers. The lollipops were not given to the children after mid-meal snacking.

Collection of saliva

For the present study, saliva collection was done twice, once before intervention (Baseline) and once post-intervention of lollipops. The person collecting the saliva was unaware of the grouping. Saliva collection was performed in all children from 1.30 pm to 3.30 pm to prevent physiological changes due to circadian rhythm. The children were instructed not to brush their teeth two hours before the procedure [15].

The children were seated on stools and with heads slightly bent forward for saliva collection. For baseline salivary status, the children were asked to chew on a tasteless paraffin piece for 5 minutes [16]. The secreted saliva was accumulated on the floor of their mouth and asked to expectorate into Eppendorf tubes. The second salivary assessment was done on the 7^th^ day after the consumption of lollipops similarly.

The saliva-containing tubes were well labeled and kept in a transport box with ice packs for transportation. On reaching the microbial laboratory, the samples were stored at -80°C till further microbial analysis was conducted.

Microbial assays and pH level determination

**Figure 3:**
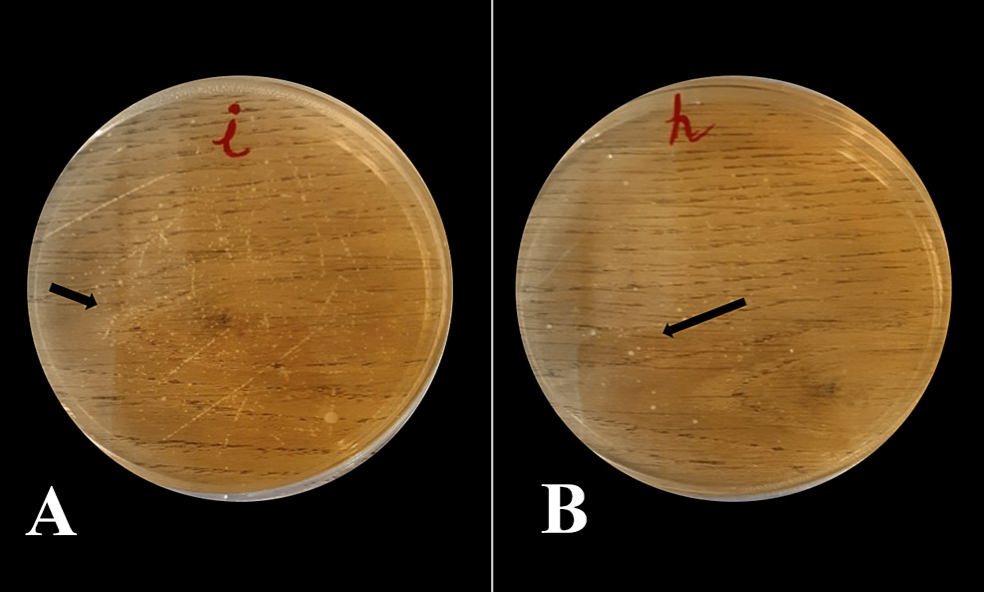
TYCSB agar plates showing counts of Streptococcus mutans in Emblica officinalis A: Pre-intervention count, B: Post-intervention count The figure is created by the author.

**Figure 4:**
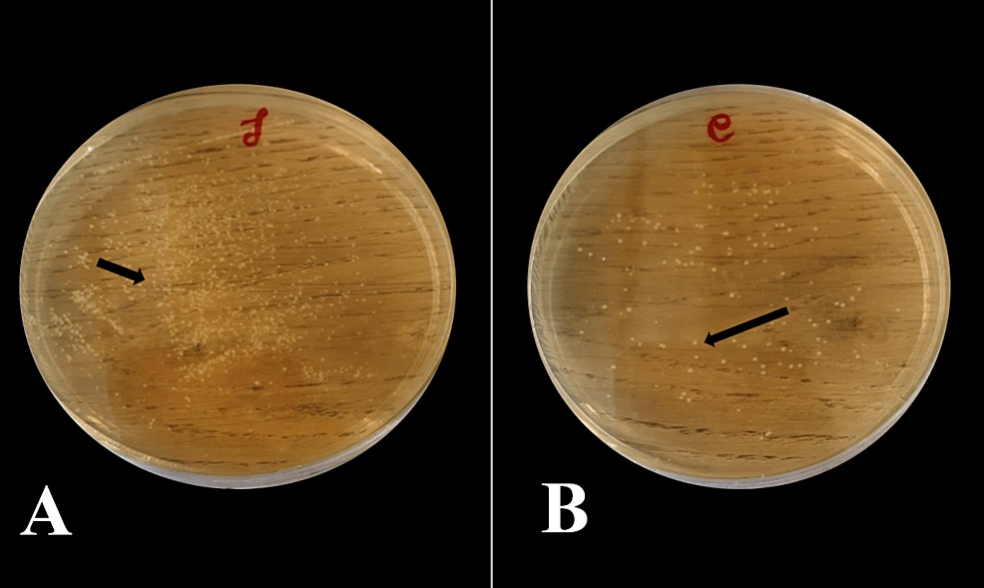
TYCSB agar plates showing counts of Streptococcus mutans in Placebo group A: Pre-intervention count, B: Post-intervention count The figure is created by the author.

The collected saliva was divided into two parts for two tests (Microbial and pH determination). For microbial analysis, one part of the sample was vortexed at 8000 g units for 15 minutes to mix all the contents adequately and serially diluted in 10-fold steps in 0.05 M phosphate buffer. The dilutions were grown into selective media for *Streptococcus mutans*, which has Tryptone yeast cysteine sucrose bacitracin (TYCSB) agar (HiMedia Laboratories Pvt Ltd, Mumbai). All plates were incubated in a Gas-Pak jar for four days at 37°C under an anaerobic environment in 7% CO2 for 72 hours [17]. The colonies were recognized by the virtue of their phenotype, and a digital colony counter (Yorco Sales Pvt Ltd, Bengaluru) was used to count the colonies, which were expressed as colony forming units (CFU) per ml. Colonies of group A pre- and post-intervention (Figure 3) and group B pre- and post-intervention were counted (Figure 4).

For pH assessment, the second part of the collected saliva was used. The pH level was assessed using a single electrode digital pH meter (Elico Ltd, Mumbai). The apparatus was precalibrated using two buffering solutions of pH 4.0 and 7.0, and the electrode tip was cleaned thoroughly using distilled water before and during the processing of each sample. The electrode was cleaned and dried with the help of sterile filter papers and then dipped into the sample. The baseline and post-intervention data were tabulated and subjected to statistical analysis.

Statistical analysis

Data were computed in an excel sheet, followed by descriptive and inferential statistics. The data was within the normal range. The normality of distribution and the homogeneity of variance for the study data was checked using the Kolmogorov-Smirnov test. The means were compared using the Student’s t-test. Students paired t-test was used for comparing the baseline and post-intervention data in both groups. Students' unpaired t-test was used to compare both the control and study group. The software used in the analysis was IBM Corp. Released 2021. IBM SPSS Statistics for Windows, Version 28.0. Armonk, NY: IBM Corp.

## Results

A total of 60 visually impaired children were included and divided into two groups in the study. In the study group, a total of 13 (43.33%) males and 17 (56.67%) females were examined, and in the control group, a total of 14 (46.67%) males and 16 (53.33%) females were examined. The gender-wise specifications of children are presented in Table 1.

**Table 1:** Gender-wise distribution of visually impaired children NS: statistically not significant. *p < .05: statistically significant. **p < .001: statistically highly significant.

Gender	Study Group (Emblica officinalis lollipop)	Control Group (Placebo lollipop)	χ2-value
Male	13 (43.33%)	14 (46.67%)	0.06 p=0.79, NS
Female	17 (56.67%)	16 (53.33%)	
Total	30 (100%)	30 (100%)	

Table 2 shows that at baseline, *Streptococcus mutans* count for the study group was 45.40±7.92 × 10^3 ^CFU/ml, which was significantly reduced to 34.53±9.72 × 10^3 ^posts seven days of intervention (t=9.06, p=0.0001). The mean *Streptococcus mutans* count for the control group was 40.70±8.14 × 10^3^ CFU/ml, followed by a significant reduction to 39.66±8.44 × 10^3^ CFU/ml after seven days of consumption (p=0.013, t=2.64, S).

**Table 2:** Comparison of mean CFU/ml of Streptococcus mutans before and after consumption of Emblica officinalis lollipop (Study group) and Placebo lollipops (Control group) respectively by using Student’s paired t-test. NS: statistically not significant. *p < .05: statistically significant. **p < .001: statistically highly significant.

	Study Parameters	Mean	N	Std. Deviation	Std. Error Mean	Percentage Reduction	t-value
Study Group	Before treatment	45.40	30	7.92	1.44	30.65%	9.06 p=0.0001**,S
	After treatment	34.53	30	9.72	1.77		
Control Group	Before treatment	40.70	30	8.14	1.48	5.90%	2.64 p=0.013*,S
	After treatment	39.66	30	8.44	1.54		

Comparing both the groups using an unpaired t-test, the study group showed a statistically significant reduction in the *Streptococcus mutans* counts after seven days (t=2.18, p=0.033, S) (Table 3).

**Table 3:** Comparison of mean CFU/ml of Streptococcus mutans after consumption of Emblica officinalis (Study group) and Placebo lollipop (Control group) by using Student’s unpaired t-test. NS: statistically not significant. *p < .05: statistically significant. **p < .001: statistically highly significant.

Groups	N	Mean	Std. Deviation	Std. Error Mean	t-value
Study Group	30	34.53	9.72	1.77	2.18 p=0.033*,S
Control Group	30	39.66	8.44	1.54	

Table 4 shows the study group’s pH scores at baseline and after seven days post-consumption to be 6.24±0.35 and 6.44±0.39, respectively. There was a statistically significant increase in the pH level (t=2.29, p=0.029). The pH score for the control group was 6.29±0.35 and 6.43±0.26 at baseline and after seven days, respectively, and was found to be statistically not significant (t=1.86, p=0.073).

**Table 4:** Comparison of mean salivary pH levels before and after consumption of Emblica officinalis lollipop (Study group) and Placebo lollipops (Control group), respectively, by using Student’s paired t-test. NS: statistically not significant. *p < .05: statistically significant. **p < .001: statistically highly significant.

	Study Parameters	Mean	N	Std. Deviation	Std. Error Mean	Percentage Reduction	t-value
Study Group	Before treatment	6.24	30	0.35	0.06	3.20%	2.29 p=0.029*,S
	After treatment	6.44	30	0.39	0.07		
Control Group	Before treatment	6.29	30	0.35	0.06	2.22%	1.86 p=0.073,NS
	After treatment	6.43	30	0.26	0.04		

On intergroup comparison, no statistically significant difference was found (t=0.17, p=0.85) (Table 5).

**Table 5:** Comparison of mean salivary pH levels after consumption of Emblica officinalis lollipop (Study group) and Placebo lollipop (Control group) by using Student’s unpaired t-test. NS: statistically not significant. *p < .05: statistically significant. **p < .001: statistically highly significant.

Groups	N	Mean	Std. Deviation	Std. Error Mean	t-value
Study Group	30	6.44	0.39	0.07	0.17 p=0.85, NS
Control Group	30	6.43	0.26	0.04	

## Discussion

According to the World Health Organization (WHO), visual impairment is defined "as a visual acuity of less than 3/60, or a corresponding visual field loss to less than 10° in the better eye with the best possible correction". Individuals with visual impairment are classified into seven levels, namely, mild, moderate, severe, very severe, total blindness, and unspecified [18]. Visual impairment can be either congenital or acquired and has many causes. Literature suggests that irrespective of congenital or acquired, children with total blindness have inferior oral hygiene practices and hence, a higher prevalence of dental caries and periodontal diseases [19]. In developing countries, a majority of special children reside in institutionalised facilities. These institutionalised visually impaired children receive a similar diet and follow the same oral hygiene practices thus they were included in the study to maintain uniformity.

Dental caries have a multifactorial aetiology and are initiated mainly by *Streptococcus mutans* amongst the other group of microorganisms [20]. Various mechanical and chemical modalities have been employed with the aim to reduce its colonization, thus reducing the dental caries formation rate [21,22]. Mechanical aids cannot be utilized to their fullest abilities by visually impaired children due to their lack of hand-eye synchronization. Antimicrobial agents such as chlorhexidine mouthwash have proven effective in reducing the *Streptococcus mutans* count but are avoided in visually impaired children due to accidental ingestion and poor psychomotor development [23]. Additionally, literature also suggests that herbal chewing gums and candies help increase the salivary flow rate, pH and thus reduce the microbial colonization of the oral cavity [24]. But chewing gums, gummies, or hard sticky candies are relatively contraindicated in children due to the high rate of aspiration and choking incidence.

Traditionally, medicinal herbs and plant extracts were known to have antimicrobial properties with relatively negligible toxic or adverse side effects [25]. *Emblica officinalis *extracts have been extensively investigated, and their antibacterial, antifungal, and antiviral properties are well-known and documented [26]. Many in-vitro studies revealed its anti-inflammatory and antioxidant properties, along with its antibacterial properties to inhibit *Streptococcus mutans *and *Lactobacillus *[14]. These medicinal effects are due to chemical constituents such as phenols, tannins, polyphenols, and flavonoids [27]. Being a component of Triphala (a powerful herbal remedy that consists of Haritaki, Bibhitaki, and Amla), it is also used in mouthwashes, irrigants, medicaments, obturating material, and toothpaste. Owing to its medicinal properties, it is also available commercially in the form of beverages, immunity boosters, or dietary supplements. Herbal lollipop has recently emerged as a beneficial vector for drug delivery. These herbal lollipops are sugar-free, and the candy is attached to a stick, thus decreasing the chances of aspiration. The lollipops can be made in different shapes, thus attracting visually impaired children to accept them. In the present study,* Emblica officinalis* extract was used to make sugar-free lollipops for the study group.

The current study evaluated the antimicrobial effect of *Emblica officinalis* lollipop on the *Streptococcus mutans* in visually impaired children. The results show that a nearly 30.65% reduction in *Streptococcus mutans* count was obtained at the end of seven days in the *Emblica officinalis* group, while it was only 5.90% in the placebo group. The reduction was statistically significant in the *Streptococcus mutans* group. Similar results were obtained in the study conducted by Velmurugan A et al. in which *Emblica officinalis* mouthwash showed a reduction in *Streptococcus mutans* count [13]. Even though both the current and previous studies adopted different age groups, the general conclusion regarding the effectiveness of *Emblica officinalis *can be made.

The study also evaluated the efficacy of *Emblica officinalis* lollipop on the pH level and showed a significant increase in only the study group. These results are comparable with a study conducted by Gao Q et al. who suggested that Amla chewing gums significantly decreased the salivary pH levels to 6.2, initially followed by a fast recovery to pH 6.9-7.0 within 15 minutes [24]. Similarly, Velmurugan A et al. showed a marginal increase in pH values when rinsed with *Emblica officinalis *mouthwash from 5.76 to 6.94 after 15 minutes [13].

The study group’s *Streptococcus mutans* count reduction could be due to the antibacterial, antifungal, antiviral, and antioxidant activity of *Emblica officinalis*. The antibacterial property is due to these active components of *Emblica officinalis* which bind to the bacterial cell wall proteins leading to a reduction in the hydrophobic adherence of *Streptococcus mutans* to the tooth surface [28]. In the placebo group, the microbial decline could be due to the sucking process of the lollipop. This oral muscular activity stimulates the salivary flow and increases the oral bicarbonate levels; this salivary flow has a direct relationship to flushing the microorganisms and reducing their oral count [29].

Following study completion, no surface irregularities were found, but a long-term ex-vitro study can be conducted to rule out the chances of *Emblica officinalis *lollipop causing surface erosions. The etiology of caries is not only restricted to microorganisms but is also related to carbohydrate retention time and oral cavity clearance due to saliva. The salivary clearance depends on the amount of ingested foodstuff, physiological factors like salivary flow rate, and saliva volume in the mouth before and after swallowing [30]. Increased salivary flow can also lead to changes in the salivary pH and the removal of oral microbes [31]. Accordingly, further research is needed on the salivary flow rate and its ionic contents in a larger group. *Emblica officinalis *has also been shown to reduce gingival inflammation, but the major limitations of the study were that the gingival and periodontal parameters weren’t assessed [32].

## Conclusions

Within the limitation of the present study, we may conclude that the *Emblica officinalis* lollipops are effective against the *Streptococcus mutans* count when compared with the placebo lollipop, while both showed a marginal change in the pH change of saliva. A modality that is most acceptable to visually impaired children should be chosen along with a beneficial drug formulation that won’t arise any pharmaceutical concerns. Therefore, the herbal formulation most commonly accepted in Indian households having an antimicrobial effect, *Emblica officinalis* was incorporated into lollipops. Further studies with bigger sample sizes, longer follow-up periods along with additional parameters like salivary flow rate and the buffering capacity of saliva are highly recommended.
